# Molecular Profiling of Thymoma and Thymic Carcinoma: Genetic Differences and Potential Novel Therapeutic Targets

**DOI:** 10.1007/s12253-016-0144-8

**Published:** 2016-11-14

**Authors:** Franz Enkner, Bettina Pichlhöfer, Alexandru Teodor Zaharie, Milica Krunic, Tina Maria Holper, Stefan Janik, Bernhard Moser, Karin Schlangen, Barbara Neudert, Karin Walter, Brigitte Migschitz, Leonhard Müllauer

**Affiliations:** 1 Department of Pathology, Medical University Vienna, Währinger Gürtel 18-20, 1090 Vienna, Austria; 2 Center for Integrative Bioinformatics Vienna, Max F. Perutz Laboratories, University of Vienna and Medical University Vienna, Vienna, Austria; 3 Department of Thoracic Surgery, Medical University Vienna, Währinger Gürtel 18-20, 1090 Vienna, Austria; 4 Center for Medical Statistics, Informatics, and Intelligent Systems, Section for Biosimulation and Bioinformatic, Medical University Vienna, Währinger Gürtel 18-20, 1090 Vienna, Austria

**Keywords:** Thymoma, Thymic carcinoma, Mutation, miRNA, Immunohistochemistry

## Abstract

Thymoma and thymic carcinoma are thymic epithelial tumors (TETs). We performed a molecular profiling to investigate the pathogenesis of TETs and identify novel targets for therapy. We analyzed 37 thymomas (18 type A, 19 type B3) and 35 thymic carcinomas. The sequencing of 50 genes detected nonsynonymous mutations in 16 carcinomas affecting ALK, ATM, CDKN2A, ERBB4, FGFR3, KIT, NRAS and TP53. Only two B3 thymomas had a mutation in noncoding regions of the SMARCB1 and STK11 gene respectively. Three type A thymomas harbored a nonsynonymous HRAS mutation. Fluorescence in situ hybridization detected in 38 % of carcinomas a CDKN2A, in 32 % a TP53 and in 8 % an ATM gene deletion, whereas only one B3 thymoma exhibited a CDKNA deletion, and none of the type A thymomas showed a gene loss. Sequencing of the total miRNA pool of 5 type A thymomas and 5 thymic carcinomas identified the C19MC miRNA cluster as highly expressed in type A thymomas, but completely silenced in thymic carcinomas. Furthermore, the miRNA cluster C14MC was downregulated in thymic carcinomas. Among non-clustered miRNAs, the upregulation of miR-21, miR-9-3 and miR-375 and the downregulation of miR-34b, miR-34c, miR-130a and miR-195 in thymic carcinomas were most significant. The expression of ALK, HER2, HER3, MET, phospho-mTOR, p16^INK4A^, PDGFRA, PDGFRB, PD-L1, PTEN and ROS1 was investigated by immunohistochemistry. PDGFRA was increased in thymic carcinomas and PD-L1 in B3 thymomas and thymic carcinomas. In summary, our results reveal genetic differences between thymomas and thymic carcinomas and suggest potential novel targets for therapy.

**Electronic supplementary material:**

The online version of this article (doi:10.1007/s12253-016-0144-8) contains supplementary material, which is available to authorized users.

## Introduction

Thymic epithelial tumors (TETs) are rare mediastinal tumors. The WHO classification distinguishes type A, AB, B1, B2 and B3 thymomas and rare other subtypes from thymic carcinomas [1]. Type A and AB thymomas are mostly benign, whereas type B1, B2 and B3 thymomas are more aggressive, with B3 thymomas having the greatest tendency for mostly intrathoracic spread. Thymic carcinoma on the contrary is a highly aggressive tumor with frequent lymphatic and hematogenous metastasis.

The therapy is based on surgery and in cases of spread or incomplete resection on chemo- and radiotherapy [2]. The development of molecularly targeted drugs has so far been limited by the lack of information on the molecular alterations of TETs and the rarity of the disease. Mutation of the tyrosine kinase KIT was the only known targetable alteration in thymic carcinoma, but it is present in only 6–12 % of cases [3, 4]. Recently, whole exome and targeted gene panel sequencing of TETs identified a specific missense mutation in GTF2I in type A thymomas and common mutations in TP53 and epigenetic regulatory genes in thymic carcinomas [5–8].

We performed a molecular profiling study to derive further insight into the pathogenesis of TETs and to identify potential novel targets for therapy. We focused the analysis on B3 thymomas and thymic carcinomas, because of their aggressiveness and due to the need to improve therapy. The analysis of type A thymomas served for comparison to elucidate molecular alterations that may be associated with aggressivenes. An additional genetic analysis of subtypes AB, B1 and B2 would have been hampered by the abundant presence of normal thymocytes in these subtypes, which impedes mutation detection and miRNA profiling.

We carried out DNA sequencing of type A and B3 thymomas and thymic carcinomas with a panel of 50 genes comprising oncogenes and tumor suppressor genes known to be frequently altered in various tumors. Currently, such gene panels are increasingly utilized in diagnostic molecular pathology for the identification of therapeutic targets in various malignancies. Such a panel sequencing is feasible with formalin fixed, paraffin embedded tissue, which is not well suited for exome sequencing, which in turn requires frozen tissue that is often not available in routine histopathology diagnostics. We complemented the genetic tumor profiling with sequencing the total miRNA pool of 5 type A thymomas and 5 thymic carcinomas for which unfixed, frozen tissue was available. Furthermore, we explored the thymomas and thymic carcinomas with a panel of immunohistochemical stains for antigens (ALK, HER2, HER3, MET, phospho-mTOR, p16^INK4A^, PDGFRA, PDGFRB, PD-L1, PTEN, and ROS1) that might constitute putative targets for therapy and fluorescence in situ hybridization for ALK, ATM, CDKN2A, FGFR3 and TP53, to detect rearrangements and/or numerical aberrations of these genes.

## Materials and Methods

### Tissue Samples

Formalin fixed, paraffin embedded type A (*n* = 18) and B3 (*n* = 19) thymoma, thymic carcinoma (*n* = 35; 34 squamous cell carcinomas, 1 lymphoepithelioma-like carcinoma) and non-neoplastic thymus (*n* = 6) tissues were retrieved from the archive of the Department of Pathology, Medical University Vienna. For miRNA sequencing unfixed frozen tissues of 5 type A thymomas and 5 thymic squamous cell carcinomas stored in liquid nitrogen were utilized. The tumors were diagnosed and subtyped according to the WHO classification [1]. The study was approved by the ethics commitee of the Medical University Vienna (projects 1167/15, 1259/15 and 1794/15).

### Cancer Gene Panel Sequencing

DNA was extracted from paraffin embedded tissue blocks with a QIAamp Tissue Kit^™^ (Qiagen, Hilden, Germany). 10 ng DNA per sample were utilized for sequencing. The DNA library was generated by multiplex polymerase chain reaction (PCR) with the Ion AmpliSeq Cancer Hotspot Panel v2^™^ (Life Technologies, Carlsbad, CA). This panel covers mutation hotspots of 50 genes, mostly oncogenes and tumor suppressor genes that are frequently mutated in tumors. Template preparation was carried out by emulsion PCR with Ion One Touch^™^ or Ion Chef^™^ instruments (Life Technologies). Sequencing was performed with an Ion Torrent PGM^™^ (Life Technologies). Sequencing data were analyzed using Variant Caller^™^, Ion Reporter^™^ (both from Life Technologies) and the VARIFI software developed by the Center for Integrative Bioinformatics Vienna. Nonsynonymous mutations detected with the Ion Torrent PGM^™^ were verified by capillary sequencing. PCR primers flanking the DNA mutation were designed. The DNA was amplified by PCR with Jump Start^™^ REDtaq^R^ Ready Mix^™^ (Sigma-Aldrich, Vienna, Austria). PCR products were cleaned using ExoSAP-IT (Affymetrix, Santa Clara, CA). The sequencing of PCR products was carried out with the BigDye^R^ Terminator v1.1 Cycle Sequencing Kit (Applied Biosystems, Waltham, MA). The resulting DNA fragments were purified with the DyeEx 96 Kit (Qiagen) and sequenced with a 3500 Genetic Analyzer (Applied Biosystems). For sequence analysis we employed the SeqScape Version 2.7 software (Applied Biosystems). In TETs with nonsynonymous mutations detected by panel and capillary sequencing DNA from normal tissue of the patients was also sequenced to exclude rare polymorphisms.

### miRNA Sequencing

MiRNA was isolated from unfixed, frozen type A thymoma and thymic carcinoma tissues with a PureLink™ miRNA Isolation Kit (Invitrogen, Carlsbad, CA). The miRNA concentration was determined using a Qubit^R^ microRNA assay kit and a Qubit^R^ fluorometer (Thermo Fisher, Waltham, MA). The size distribution of the miRNAs was validated with an Agilent small RNA kit and an Agilent Bioanalyzer instrument (Agilent, Santa Clara, CA). miRNA libraries for sequencing were generated with a Ion Total RNA Seq Kit (Thermo Fisher). Template preparation was achieved with the Ion PGM^™^ IC 200 Kit on an Ion Chef instrument (Thermo Fisher). Sequencing was performed using the Ion PGM™ Sequencing 200 Kit on an Ion Torrent PGM with Ion 316 chips (Thermo Fisher). An initial adapter trimming of sequencing reads was performed with the Torrent Suite™ software (Thermo Fisher). The sequences were then converted to fastq format and exported for further analysis in the CLC™ Genomics Workbench 8.5 (Qiagen, Hilden, Germany). As recommended by the manufacturer, a second adapter trimming was performed, followed by quality trimming, and mapping to miRBase v21. Quality control of these fastq files was conducted using FastQC. To identify possible RNA degradation, the reads were also mapped against human rRNA and tRNA sequences.

### Fluorescence In Situ Hybridization (FISH)

FISH was performed with 4 μm thin sections of type A and B3 thymoma and thymic carcinoma tissue microrrays. The arrays were generated with a manual tissue arrayer from Beecher Instruments (Sun Prairie, WI). Three tissue cores 0.8 mm in diameter each were taken per tumor. The following FISH probes were employed: ALK (2p23.1; Abbott, Abbott Park, IL), ATM (11q22), CDKN2A (9p21)/Centromer 9, FGFR3 (4p16), PTEN (10q23.31)/Centromer 10, TP53 (17p13)/Centromer 17 (Metasystems, Altlussheim, Germany) and ROS1 (Zytovision, Bremerhaven, Germany). 200 cell nuclei per tumor were evaluated. A hetero-/homozygous gene deletion or aneuploidy was concluded when more than 25 % of nuclei showed relevant aberrant fluorescence signals.

### Immunohistochemistry

Immunohistochemistry was performed using tissue arrays of type A and B3 thymomas and thymic carcinomas. 2 μm sections of the tissue arrays were stained on a Ventana Benchmark Ultra (Ventana, Tucson, AZ) with extended heat-induced epitope retrieval with CC1 buffer and the ultraView Universal DAB Detection Kit (Ventana). The following antibodies were employed: ALK (clone 1A4; Zytomed, Berlin, Germany), HER2 (clone 4B5; Ventana), HER3 (clone SP71; Abcam, Milton, UK), MET (clone SP44; Ventana), phospho-mTOR (clone 49F9; Cell Signalling, Danvers, MS), p16^INK4A^ (clone E6H4; Ventana), PDGFRA (rabbit polyclonal; Thermo Fisher Scientific), PDGFRB (clone 28E1, Cell Signalling), PD-L1 (clone E1L3N; Cell Signalling), PTEN (clone Y184; Abcam) and ROS1 (clone D4D6; Cell Signalling). An immunohistochemial score was determined by multiplying the percentage of positive cells by their respective staining intensity (0 = negative, 1 = weak, 2 = moderate, 3 = strong). Immunohistochemical score (maximum 300) = (% negative x 0) + (% weak x 1) + (% moderate x 2) + (% strong x 3).

### Statistics

MiRNA sequencing data were analyzed in a CLC Genomics Workbench (Qiagen) with an embedded version of EdgeR [9]. To correct for multiple testing, the false discovery rate method (FDR) was used. A FDR below 0.05 was defined as differentially expressed. Graphs, plots and heatmaps were created with Microsoft Excel, CLC Genomics Workbench, and IBM SPSS Statistics 20. Immunohistochemistry results were evaluated employing the Shapiro-Wilk, Kolmogorov-Smirnoff and Kruskal-Wallis tests. Patient survival data were determined using the Kaplan-Meier estimator and significances were calculated by the log-rank and Breslow tests.

## Results

### Targeted Cancer Gene Sequencing Reveals Genetic Differences Between Thymoma and Thymic Carcinoma and Identifies Potential Novel Targets for Therapy

We sequenced mutation hotspot regions of 50 genes that are known to be frequently mutated in various human cancers. A total of 35 thymic carcinomas, 19 type B3 thymomas and 18 type A thymomas were analyzed. In 16 (46 %) thymic carcinomas a nonsynonymous mutation was detected that is predicted to alter the amino acid sequence of the encoded protein (Tables 1 and 2). The most frequently altered gene was TP53 which was mutated in 9 carcinomas, with one case harboring two TP53 mutations. Four carcinomas exhibited a missense mutation in the tumor suppressor CDKN2A, which was thus the second most frequently mutated gene. Two carcinomas each harbored a missense mutation in the fibroblast growth factor receptor 3 (FGFR3) and the receptor tyrosine kinase KIT. The receptor tyrosine kinases ALK and ERBB4, the serine/threonine kinase ATM, and the GTPase NRAS were mutated in one thymic carcinoma each (Tables 1 and 2).

**Table 1:** Genes mutated in type A and B3 thymomas and thymic carcinomas

	Mutated genes	Number of mutated cases
Type A thymoma (*n* = 18)	HRAS	3
Type B3 thymoma (*n* = 19)	SMARCB1	1
	STK11	1
Thymic carcinoma (*n* = 35)	TP53	9
	CDKN2A	4
	FGFR3	2
	KIT	2
	ALK	1
	ATM	1
	ERBB4	1
	NRAS	1

**Table 2:** Mutations in type A and B3 thymomas and thymic carcinomas

Case	Tumor	Gene	Gene region	Chromosome position	cDNA	Protein variant	Variant frequency
1	A thymoma	HRAS	Exon 3	chr11:533874	c.182A>G	p.Q61R	46 %
2	A thymoma	HRAS	Exon 3	chr11:533874	c.182A>G	p.Q61R	59 %
3	A thymoma	HRAS	Exon 2	chr11:534276	c.47A>C	p.K16T	48 %
4	B3 thymoma	SMARCB1	3′UTR	chr22:24176384	c.1185-17C>T	unknown	55 %
5	B3 thymoma	STK11	Intron 3	chr19:1220354	c.1580 + 16_17del	unknown	59 %
6	Carcinoma	TP53	Exon 1	chr17:7578383	c. 529_546del	p.45_50delPHHERC	87 %
7	Carcinoma	TP53	Exon 8	chr17:7577085	c. 853C>T	p.E285K	50 %
8	Carcinoma	CDKN2A	Exon 2	chr9:21971029	c.329G>A	p.W110*	63 %
9	Carcinoma	CDKN2A	Exon 2	chr9:21971029	c. 329G>A	p.W110*	81 %
		FGFR3	Exon 7	chr4:1803568	c. 746C>G	p.S249C	42 %
10	Carcinoma	TP53	Exon 8	chr17:7577129	c.692T>C	p.F231S	60 %
11	Carcinoma	TP53	Exon 10	chr17:7574029	c.998G>A	p.R333H	51 %
12	Carcinoma	CDKN2A	Exon 2	chr9:21971186	c.172C>T	p.R58*	95 %
		TP53	Exon 2	chr17:7578211	c.638G>A	p.R213Q	81 %
13	Carcinoma	FGFR3	Exon 9	chr4:1806099	c.1118A>G	p.Y373C	35 %
14	Carcinoma	CDKN2A	Exon 2	chr9:21971117	c.241C>T	p.P81S	47 %
		TP53	Exon 8	chr17:7577085	c.853G>A	p.E285K	48 %
15	Carcinoma	KIT	Exon 17	chr4:55599342	c.2468A>C	p.Y823S	48 %
16	Carcinoma	ALK	Exon 25	chr2:29432664	c.3824C>T	p.R1275Q	25 %
		TP53	Exon 5	chr17:7578457	c.473 C>T	p.R158H	49 %
17	Carcinoma	KIT	Exon 11	chr4:55593661	c.1727T>C	p.L576P	37 %
18	Carcinoma	ERBB4	Exon 9	chr2:212576901	c.998C>T	p.A333V	46 %
19	Carcinoma	NRAS	Exon 3	chr1:115256528	c.183T>A	p.Q61H	35 %
		TP53	Exon 5	chr17:7578423	c.507 C>T	p.M169I	87 %
		TP53	Exon 8	chr17:7577085	c.853 C>T	p. E285K	79 %
20	Carcinoma	ATM	Exon 8	chr11:108117798	c.1009 C>T	p.R337C	53 %
21	Carcinoma	TP53	Exon 10	chr17:7574003	c.1024C>T	p.R342*	49 %

The B3 thymomas did not harbor any of the mutations detected in thymic carcinomas. Only in two (11 %) B3 thymomas a mutation was detected (Tables 1 and 2). One was a point mutation in the 3′ untranslated region at position −17 of the tumor suppressor SMARCB1. The other represented a deletion of two nucleotides +16 and +17 upstream of the exon-intron boundary of exon 4 of the tumor suppressor STK11. At present it is unknown wether these mutations have any effect on gene function, in particular on RNA translation or RNA splicing.

Three (17 %) of the 18 type A thymomas harbored a non-synonymous mutation in the HRAS oncogene (Tables 1 and 2). One of these thymomas (case 3, Table 2) was a histomorphologically atypical variant type A thymoma [10] with a lung metastasis.

### Fluorescence In Situ Hybridization (FISH) - Frequent Loss of CDKN2A and TP53 in Thymic Carcinomas

DNA sequencing revealed TP53 and CDKN2A as the most frequently mutated genes in thymic carcinomas and detected one case of ATM mutation. We therefore performed FISH to determine wether additional deletions of these tumor suppressor genes were present. A total of 28 thymic carcinomas were analyzed for a loss of TP53. Nine (32 %) exhibited a heterozygous or mixed heterozygous/homozygous deletion of TP53 (Table 3). In 4 cases a heterozygous deletion was associated with a mutation of the remaining TP53 allele. Twelve of 32 (38 %) analyzed thymic carcinomas exhibited a loss of the CDKN2A gene, which was heterozygous in three, homozygous in four and mixed heterozygous/homozygous in five cases (Table 3). Cause specific survival, freedom of recurrence, disease free and overall survival of thymic carcinomas with a CDKN2A (Supplementary Fig. S1) or TP53 (Supplementary Fig. S2) gene alteration (mutation and/or deletion) did not differ from wild-type cases. A TP53 gene loss was not present in 17 B3 thymomas analyzed. Only in one of 17 (6 %) B3 thymomas a mixed heterozygous/homozygous CDKN2A gene deletion was detected. In type A thymomas, no TP53 and CDKN2A deletions were present (Table 3). A heterozygous deletion of ATM was noted in two (8 %) of 26 thymic carcinomas. None of them harbored an ATM mutation by gene sequencing. In type A and B3 thymomas no ATM loss was found (Table 3).

**Table 3:** Fluorescence in situ hybridization. ATM, CDKN2A and TP53 gene deletions in type A and B3 thymomas and thymic carcinomas

Tumor	Gene	Heterozygous deletion	Homozygous deletion	Mixed heterozygous/homozygous deletion	% cases with a deletion
A thymoma (*n* = 15)	ATM	0	0	0	0 %
B3 thymoma (*n* = 17)	ATM	0	0	0	0 %
Thymic carcinoma (*n* = 26)	ATM	2	0	0	8 %
A thymoma (*n* = 14)	CDKN2A	0	0	0	0 %
B3 thymoma (*n* = 17)	CDKN2A	0	0	1	6 %
Thymic carcinoma (*n* = 32)	CDKN2A	3	4	5	38 %
A thymoma (*n* = 15)	TP53	0	0	0	0 %
B3 thymoma (*n* = 17)	TP53	0	0	0	0 %
Thymic carcinoma (*n* = 28)	TP53	8	0	1	32 %

Motivated by the two thymic carcinomas with an FGFR3 mutation and the one carcinoma with an ALK mutation, we also performed FISH for these two genes. However a FGFR3 amplification or ALK translocation was not detected in 25 and 27 evaluated thymic carcinomas, respectively (data not shown).

### miRNA Sequencing Identifies Differential miRNA Expression in Type A Thymoma and Thymic Carcinoma

**Fig. 1:**
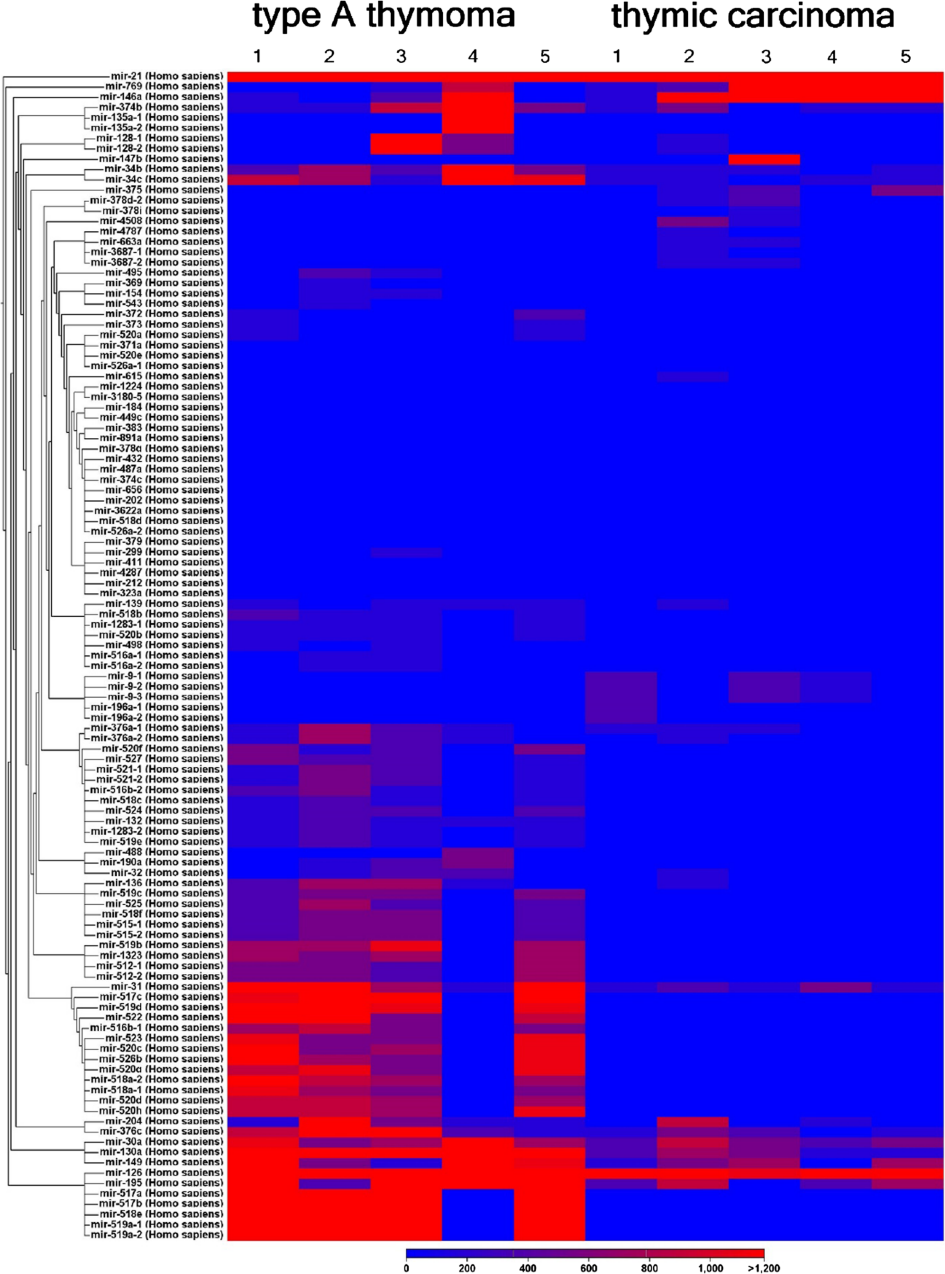
Heatmap of all miRNA transcripts differentially expressed in type A thymomas and thymic carcinomas at a false discovery rate p-value < 0.05

**Fig. 2:**
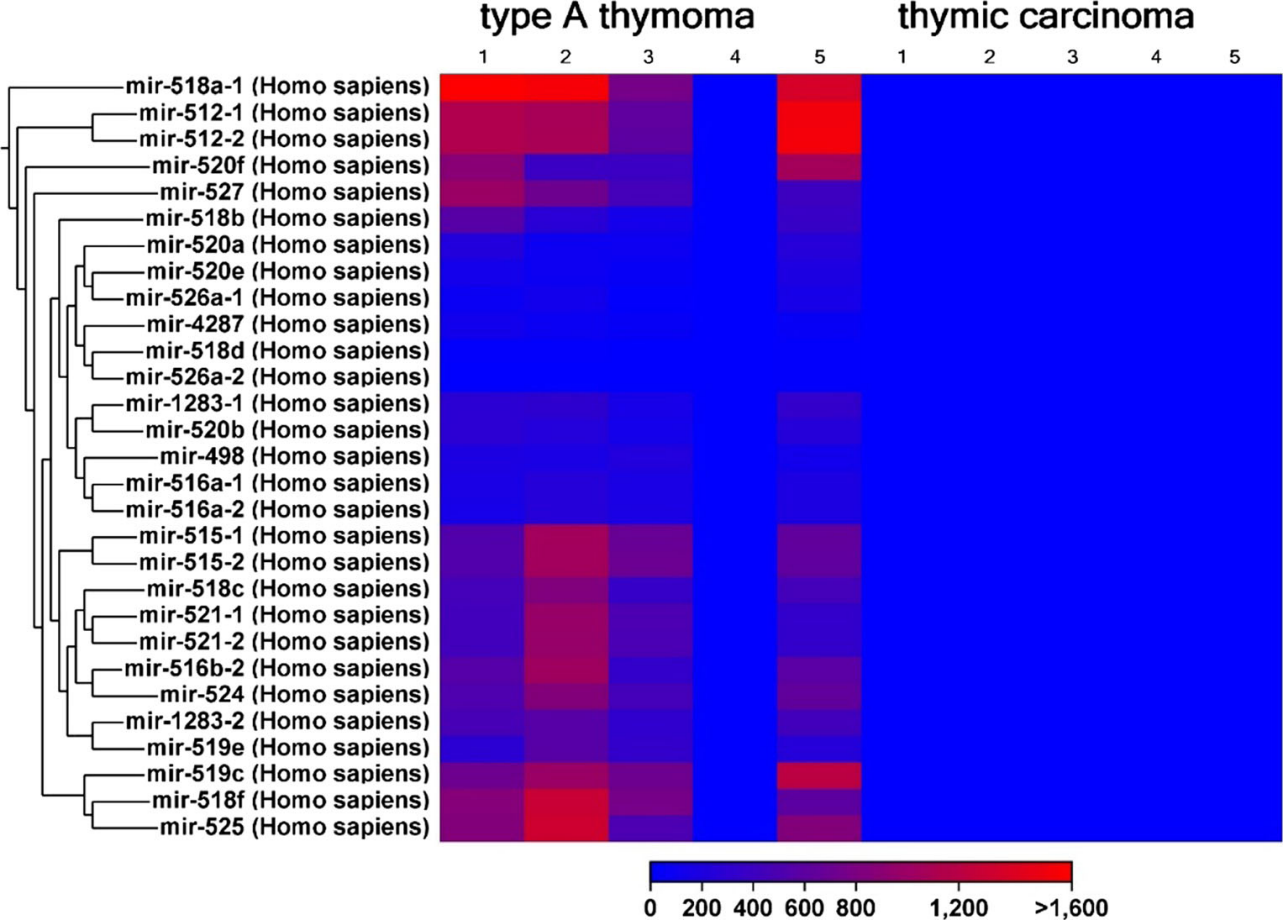
Heatmap showing high C19MC miRNA cluster expression in four of five type A thymomas, but virtually no expression in thymic carcinomas at a false discovery rate p-value < 0.0005

**Fig. 3:**
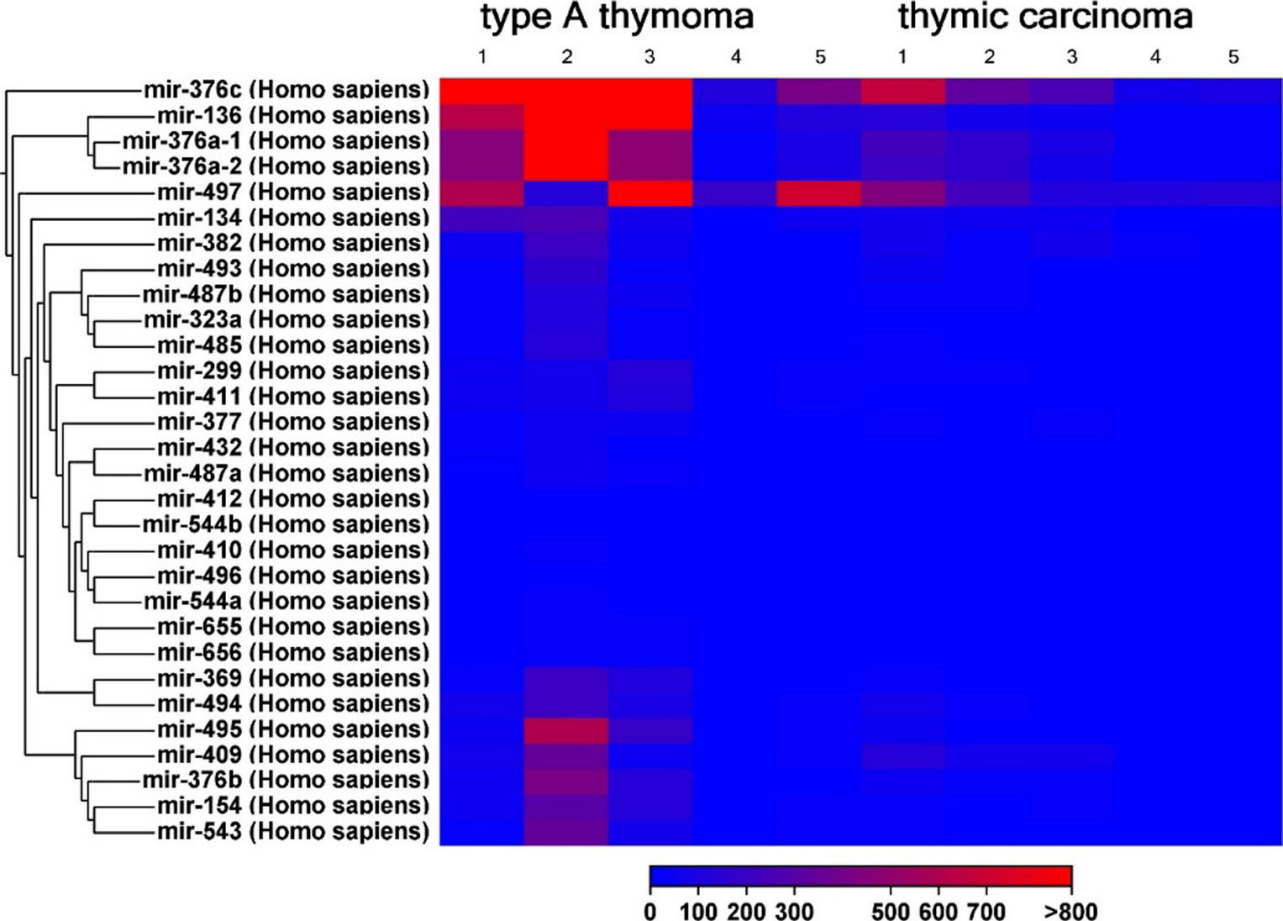
Heatmap showing different expression of C14MC miRNA cluster members in type A thymomas and thymic carcinomas at a false discovery rate p-value < 0.05

**Fig. 4:**
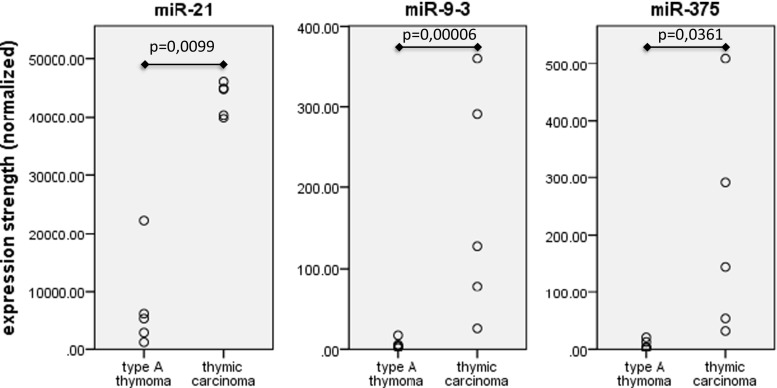
Non-clustered miRNAs with stronger expression in thymic carcinomas than in type A thymomas

**Fig. 5:**
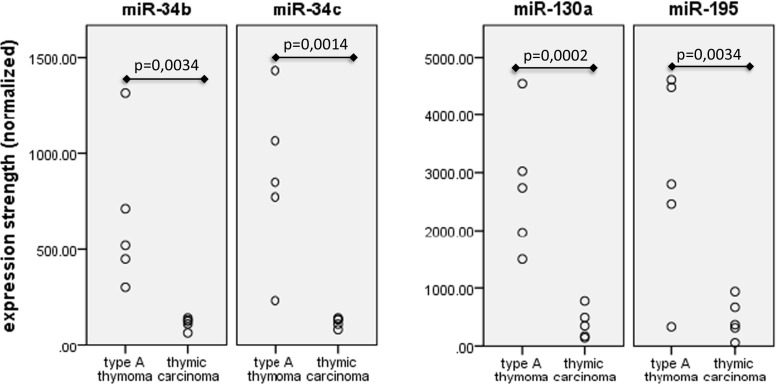
Non-clustered miRNAs with lower expression in thymic carcinomas than in type A thymomas

The expression of 1218 different miRNAs was detected in type A thymomas and thymic carcinomas. 113 of the miRNAs were differentially expressed between the two tumor entities at a false discovery rate corrected p-value of below 0.05 (Fig. 1). Sixtythree of these 113 miRNAs were part of two large imprinted miRNA clusters on chr19q13.4 and chr14q32, called C19MC and C14MC, respectively. C19MC miRNAs were highly expressed in 4 of the 5 type A thymomas and completely silenced in all 5 thymic carcinomas (Fig. 2). C14MC transcripts were also significantly downregulated in thymic carcinomas, but the cluster was not completely silenced (Fig. 3). Fifty of the differentially expressed miRNAs were not clustered. Amongst them, most significant was the strong expression of miR21, miR-9-3 and miR-375 in thymic carcinomas and their very low abundance in type A thymomas (Fig. 4). On the contrary miR-34b, miR-34c, miR-130a and miR-195 were of low abundance in thymic carcinomas, but strongly expressed in type A thymomas (Fig. 5).

### Immunhistology Identifies PDGFRA and PD-L1 as Potential Therapeutic Targets in Thymic Carcinoma and B3 Thymoma

Type A and B3 thymoma and thymic carcinoma tissues were stained with antibodies to ALK, HER2, HER3, MET, phospho-mTOR, PDGFRA, PDGFRB, PD-L1, p16^INK4A^, PTEN and ROS1 to identify potential targets for therapy.

The tumor cells did not express ALK, HER2, and HER3 (Table 4).

**Table 4:** Immunohistochemical expression of putative therapeutic targets in type A and B3 thymomas and thymic carcinomas

	ALK	HER2	HER3	MET	mTOR	PDGFRA	PDGFRB	PD-L1	P16^INK4A^	PTEN	ROS1
Type A (*n* = 14–15)											
absent	15 (100 %)	15 (100 %)	15 (100 %)	13 (87 %)	0 (0 %)	0 (0 %)	9 (60 %)	13 (87 %)	5 (33 %)	0 (0 %)	12 (80 %)
low	0 (0 %)	0 (0 %)	0 (0 %)	2 (13 %)	12 (80 %)	4 (29 %)	6 (40 %)	1 (6.5 %)	7 (47 %)	11 (73 %)	3 (20 %)
intermediate	0 (0 %)	0 (0 %)	0 (0 %)	0 (0 %)	3 (20 %)	10 (71 %)	0 (0 %)	1 (6.5 %)	2 (13 %)	4 (27 %)	0 (0 %)
high	0 (0 %)	0 (0 %)	0 (0 %)	0 (0 %)	0 (0 %)	0 (0 %)	0 (0 %)	0 (0 %)	1 (7 %)	0 (0 %)	0 (0 %)
Type B3 (*n* = 16–17)											
absent	17 (100 %)	17 (100 %)	17 (100 %)	17 (100 %)	7 (41 %)	0 (0 %)	14 (88 %)	4 (24 %)	16 (94 %)	1 (6 %)	17 (100 %)
low	0 (0 %)	0 (0 %)	0 (0 %)	0 (0 %)	7 (41 %)	5 (29 %)	2 (12 %)	7 (41 %)	1 (6 %)	2 (12 %)	0 (0 %)
intermediate	0 (0 %)	0 (0 %)	0 (0 %)	0 (0 %)	3 (18 %)	7 (41 %)	0 (0 %)	6 (35 %)	0 (0 %)	5 (29 %)	0 (0 %)
high	0 (0 %)	0 (0 %)	0 (0 %)	0 (0 %)	0 (0 %)	5 (29 %)	0 (0 %)	0 (0 %)	0 (0 %)	9 (53 %)	0 (0 %)
Carcinoma (*n* = 29–31)											
absent	31 (100 %)	31 (100 %)	31 (100 %)	31 (100 %)	9 (31 %)	0 (0 %)	31 (100 %)	14 (47 %)	16 (53 %)	1 (3 %)	31 (100 %)
low	0 (0 %)	0 (0 %)	0 (0 %)	0 (0 %)	12 (41 %)	1 (3 %)	0 (0 %)	11 (37 %)	5 (17 %)	6 (19 %)	0 (0 %)
intermediate	0 (0 %)	0 (0 %)	0 (0 %)	0 (0 %)	4 (14 %)	3 (10 %)	0 (0 %)	4 (13 %)	3 (10 %)	10 (32 %)	0 (0 %)
high	0 (0 %)	0 (0 %)	0 (0 %)	0 (0 %)	4 (14 %)	27 (87 %)	0 (0 %)	1 (3 %)	6 (20 %)	14 (45 %)	0 (0 %)

A weak MET expression was noted in only two (13 %) of 15 type A thymomas. B3 thymomas and thymic carcinomas did not express MET.

The expression of phospho-mTOR was similar in type A and B3 thymomas and thymic carcinomas (Table 4).

**Fig. 6:**
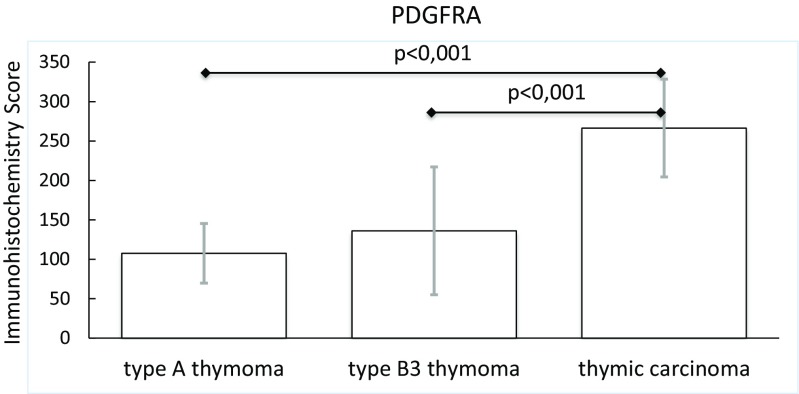
PDGFRA protein expression in type A and B3 thymomas and thymic carcinomas

The expression of PDGFRA was significantly increased in thymic carcinomas as compared to type A and B3 thymomas (Table 4, Fig. 6, Supplementary Fig. S3). A low PDGFRB expression was noted in 40 % of type A and 12 % of B3 thymomas. Thymic carcinomas were PDGFRB negative (Table 4).

**Fig. 7:**
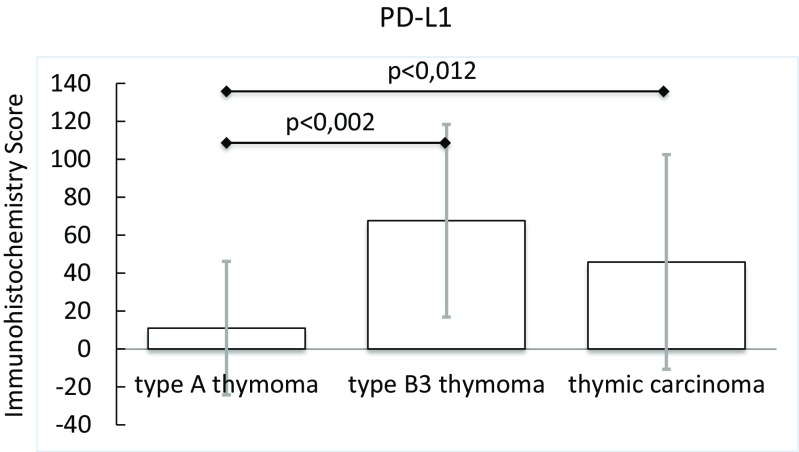
PD-L1 protein expression in type A and B3 thymomas and thymic carcinomas

The immune check point inhibitor PD-L1 was expressed in only 2 (13 %) of 15 type A thymomas, but 13 (76 %) of 17 B3 thymomas and 16 (53 %) of 30 thymic carcinomas (Table 4, Fig. 7, Supplementary Fig. S4). The expression of PD-L1 in thymic carcinomas did not correlate with a difference in cause specific survival, freedom of recurrence, disease free and overall survival (Supplementary Fig. S5).

**Fig. 8:**
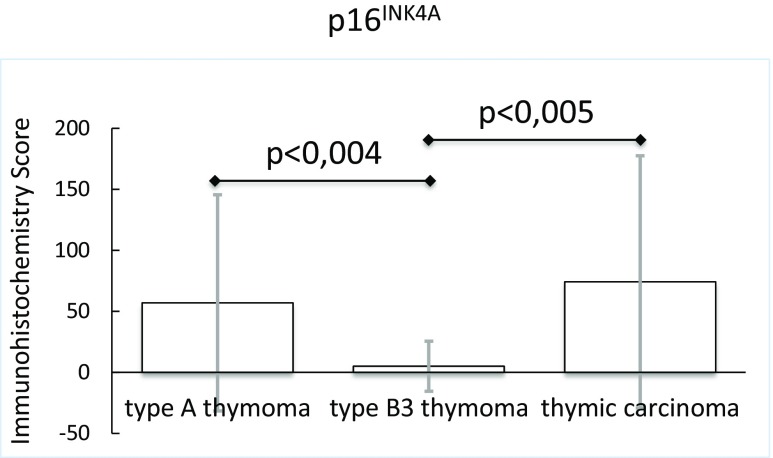
p16^INK4A^ protein expression in type A and B3 thymomas and thymic carcinomas

In six non-neoplastic thymi of adult individuals, analyzed for comparison with TETs, p16^INK4A^ expressing epithelial cells were scarce and located in close vicinity to Hassall’s corpuscles (data not shown). In TETs, 10 (67 %) of 15 type A thymomas and 14 (47 %) of 30 thymic carcinomas expressed p16^INK4A^, wheras only one of 17 (6 %) B3 thymomas exhibited a low p16^INK4A^ positivity (Table 4, Fig. 8). In thymic carcinomas, but not in type A and B3 thymomas, a lack of p16^INK4A^ expression was largely associated with CDKN2A mutation or gene deletion (data not shown). The expression of p16^INK4A^ did not correlate with a difference in cause specific survival, freedom of recurrence, disease free and overall survival in thymic carcinomas (Supplementary Fig. S6).

The expression of PTEN was heterogenous, with no significant differences between the three tumor entities (Table 4). A lack of PTEN expression was noted in one B3 thymoma and one thymic carcinoma. However, both tumors retained two PTEN signals as assessed by FISH (data not shown). Thus, the lack of PTEN expression was not caused by gene deletion, but by another mechanism, presumably PTEN promoter methylation.

A low ROS1 expression was present in 3 (20 %) of 15 type A thymomas, but none of them harbored a ROS1 gene translocation as assessed by FISH (data not shown). B3 thymomas and thymic carcinomas were ROS1 negative (Table 4).

## Discussion

By gene panel and miRNA sequencing, FISH and immunohistochemistry we found genetic differences between thymoma (type A and B3) and thymic carcinoma and identified potential novel targets for therapy.

The most frequently altered gene was the tumor suppressor CDKN2A. It encodes p16^INK4A^ and p14^ARF^ by alternative splicing. p16^INK4A^ inhibits cell cycle progression by blocking cyclin dependent kinases 4 and 6, whereas p14^ARF^ activates the TP53 tumor suppressor. A CDKN2A alteration may lead to activation of cyclin dependent kinases. Inhibitors for these kinases are currently being investigated in clinical trials for various malignancies and might constitute a therapeutic option also for thymic carcinomas [11]. In a previous work by Petrini I. et al. a lack of p16^INK4A^ protein expression identified TETs with a worse disease related survival [12]. In our study, however, CDKN2A gene loss or mutation did not correlate with a worse outcome in thymic carcinomas.

The second most frequently altered gene in thymic carcinomas was the tumor suppressor TP53. A TP53 protein overexpression, which is often caused by TP53 mutation, has been reported in a cohort of 25 thymic carcinomas to be associated with a worse disease free survival [7]. In our study with 31 evaluable thymic carcinomas TP53 gene loss or mutation was, however, not a prognostic marker for disease free or overall survival.

The tyrosine kinase KIT is the receptor for stem cell factor. It contributes to the growth and survival of tumors and is best known for its involvement in the pathogenesis of gastrointestinal stromal tumors [13]. Mutated KIT constitutes a therapeutic target for kinase inhibitors such as imatinib. KIT mutation is rare in thymic carcinoma, but so far the only known molecular target based on few, but encouraging case reports [14, 15]. In our series two carcinomas with KIT mutations that predict sensitivity to imatinib were present.

The FGFR family includes four receptor tyrosine kinases. FGFR genes are deregulated in solid tumors by amplification, translocation or mutation [16]. FGFR3 mutations are particulary frequent in bladder cancer, where they are associated with low grade, early stage, and better survival [17]. We observed a FGFR3 missense mutation in two thymic carcinomas. Thus, inhibition of FGFR3 might represent a novel target in a subset of thymic carcinomas. Several FGFR inhibitors are currently in development and being evaluated in clinical trials [16, 18].

ALK is a receptor tyrosine kinase that, when altered by chromosomal inversion, translocation, amplification or mutation, plays an oncogenic role in certain cancers. Best known are ALK gene alterations in anaplastic large cell lymphoma, lung adenocarcinoma, inflammatory myofibroblastic tumor and neuroblastoma [19]. The p.R1275Q ALK mutation observed in one of our thymic carcinomas is an activating point mutation in the kinase domain and known as one of the most common mutations in neuroblastoma [20]. We did not detect an ALK rearrangement or amplification in TETs by FISH. An ALK mutation had also been detected by Petrini I. et al. in an AB and a B3 thymoma by exome and panel sequencing [5]. Small molecule inhibitors such as crizotinib, that target the kinase activity of ALK, are established in the clinic and might be a novel therapeutic in ALK mutated TETs as well.

The epidermal growth factor receptor family includes EGFR, ERBB2 (also known as HER2), ERBB3 (also known as HER3) and ERBB4. Therapy of colon and breast cancer with anti-EGFR and anti-ERBB2 antibodies, respectively, and of EGFR mutated lung adenocarcinoma with tyrosine kinase inhibitors is well established [21]. ERBB4 mutations have been identified in lung, breast and gastric cancer and melanoma. Several of these ERBB4 mutations were shown to be oncogenic in melanoma models and could be inhibited by treatment with lapatinib [22]. ERBB4 mutated thymic carcinoma might also be inhibited by EGFR family blockers such as lapatinib and afatinib [21].

ATM is a kinase which induces cell cycle arrest and facilitates DNA repair. The inhibition of DNA repair has become an attractive strategy in cancer therapy. Small molecule inhibitors of ATM are currently in preclinical and clinical development. They may enhance susceptibility of cancer cells to DNA damaging chemotherapy. Many tumors acquire defects in DNA damage repair in order to tolerate genomic instability which is a characteristic of malignant transformation. Defects in ATM signaling are synthetically lethal with PARP inhibition, suggesting that combined inhibition of ATM and PARP may be a therapeutic strategy [23]. PARP inhibitors are extremely efficient against cancer cells bearing ATM defects, as shown in the context of mantle cell lymphoma [24, 25].

One thymic carcinoma harbored a NRAS and three type A thymomas a HRAS mutation. HRAS was the only mutated gene in type A thymomas in this study. One of the HRAS mutated cases was a histomorphologically atypical variant [10] with a lung metastasis. At present the RAS oncogenes are still not drugable targets, although multiple clinical trials targeting RAS interacting molecules or downstream signaling partners are ongoing [26, 27].

We have identified 113 miRNAs that are differently expessed in type A thymomas and thymic carcinomas. Thereov, 63 miRNAs belong to two large imprinted miRNA clusters, namely C19MC on chromosome 19q13.42 [28] and C14MC on chromosome 14q32 [29]. C19MC overexpression has been reported in embryonal pediatric brain tumors caused by fusion with TTYH1 [30] or focal genomic amplification [31]. Overexpression of this cluster is furthermore observed in thyroid and parathyroid adenomas, hepatic mesenchymal hamartomas, hepatocellular carcinomas and a subset of tamoxifen resistant breast cancers [32–35]. C19MC overexpression in type A and AB thymomas has recently been reported by Radovich M. et al., who suggested that one of the key functions of the cluster is the activation of the PI3K/AKT pathway [36]. However, a report by Ucar et al. [37] indicates that C19MC is also expressed in normal medullary and cortical thymic epithelial cells. Thus, both of the putative progenitor cell types of TETs express C19MC. We therefore propose that C19MC expression is silenced in thymic carcinomas. This may be caused by promoter methylation [28].

C14MC miRNA expression was decreased in thymic carcinomas as compared to type A thymomas, but still present to some degree, with not all miRNAs of the cluster affected. Infering from published work suggesting that C14MC functions as a large tumor suppressor cluster in GIST [38] and glioma [39] we assume that the downregulation of C14MC miRNAs might exert a tumor promoting effect in thymic carcinomas.

Among the non-clustered miRNAs with significant differences in expression between type A thymomas and thymic carcinomas, the low expression of miR-34b, miR-34c, miR-130a and miR-195 in thymic carcinomas was most pronounced. These four miRNAs are putative tumor suppressors. In non-small cell lung cancer (NSCLC) immune evasion of the tumor via PD-L1 is mediated by miR-34 [40]. A more frequent PD-L1 protein expression in thymic carcinomas as compared to type A thymomas has been observed in our study and might likewise be regulated by miR-34. miR-130a downregulation in hepatocellular carcinoma correlates with poor prognosis [41]. miR-195 prevents cell proliferation and promotes apoptosis by targeting Cyclin D1 and BCL2 and is decreased in many solid tumors [42, 43].

Among the non-clustered miRNAs overexpressed in thymic carcinoma as compared to type A thymoma miR21, miR-9-3 and miR-375 were most significant. miR21 is regarded to be oncogenic. It is upregulated in various solid tumors, lymphomas and leukemias [44, 45]. In contrast to our findings in thymic carcinomas miR-9-3 has been reported to be repressed by methylation in NSCLC [46]. miR-375 was first identified as a pancreatic islet-specific miRNA that regulated insulin secretion [47]. In malignant tumors it is often downregulated and might constitute a tumor suppressor. In breast and prostate cancer, however, it is upregulated [48]. Thus, depending on the cellular context it may exert an oncogenic influence, probably also in thymic carcinomas.

Immunohistochemistry was performed to study the expression of ALK, HER2, HER3, MET, phospho-mTOR, p16^INK4A^, PDGFRA, PDGFRB, PD-L1, PTEN and ROS1 in type A and B3 thymomas and thymic carcinomas. None of the thymomas and thymic carcinomas analyzed expressed ALK, HER2 and HER3. The lack of HER2 and HER3 expression in our samples is in contrast to previous publications. Weissferdt A. et al. reported HER2 expression in 58 % of 24 squamous thymic carcinomas. Only a single case showed HER2 gene amplification by FISH [49]. The authors furthermore described HER3 positivity in 45.8 % of the carcinomas. Pan CC. et al. detected HER2 positivity in nine of 17 thymic carcinomas, but no HER2 gene amplification could be demonstrated by FISH [50]. The reason for these discrepancies in HER2 and HER3 immunohistochemistry is not known, but might be attributable to the use of different antibodies and staining protocols. As can be seen at least from our study HER2 and HER3 do not constitute a therapeutic target in type A and B3 thymomas and thymic carcinomas.

The proto-oncogene MET is a receptor tyrosine kinase that can promote tumor development and progression. Tumors with MET overexpression, gene amplification and exon 14 skipping mutations are candidates for MET targeted therapies in clinical trials [51, 52]. A low MET protein expression was noted in only two type A thymomas, whereas B3 thymomas and thymic carcinomas did not express MET. Thus, MET does not represent a potential therapeutic target in TETs.

Aberrations in the PI3K/mTOR/AKT pathway are common in solid tumors [53]. Drugs have been developed that target different components of this pathway. We did not detect mutations in PIK3CA and AKT1 in type A and B3 thymomas or thymic carcinomas. Furthermore, the expression of phospho-mTOR protein did not differ significantly among type A and B3 thymomas and thymic carcinomas.

PDGFRA was expressed by medullary epithelial cells of fetal and postnatal normal thymus and by epithelial tumor cells in 10 analyzed thymomas [54]. A further study [55] reported PDGFRA protein expression in all 26 thymomas of various subtypes and 10 thymic carcinomas analyzed. PDGFRB protein was present in only one third of the tumors, however, it was not specified whether epithelial cells or stromal cells were positive [55]. In our study the expression of PDGFRA was increased in thymic carcinomas as compared to type A and B3 thymomas. The PDGFRA gene, however, was not mutated. A weak PDGFRB positivity was present in a few type A and B3 thymomas, whereas all thymic carcinomas were negative. An efficacy of the multi-target tyrosine kinase inhibitors sorafenib and sunitinib that block also PDGFRA has been observed in thymic carcinomas [56, 57]. However, the expression of PDGFRA has not been determined by immunohistochemistry in these studies and it is therefore not possible to conclude whether the efficacy of the drugs was due to PDGFRA inhibition or blockade of other kinases.

The expression of p16^INK4A^ protein in normal tissue is generally low. In non-neoplastic thymic tissues we have observed sparce p16^iNK4A^ positive epithelial cells in the vicinity of Hassall’s corpuscles. In neoplastic cells oncogenic stress can induce p16^INK4A^. Furthermore, tumors with loss of the RB gene or RB protein inactivation by viral oncogenic proteins harbor high levels of p16^INK4A^ [58]. Ten (67 %) of 15 type A thymomas and 14 (47 %) of 30 thymic carcinomas expressed p16^INK4A^, whereas only one (6 %) of 17 B3 thymomas was weakly p16^INK4A^ positive. In thymic carcinomas a lack of p16^INK4A^ protein expression was largely associated with CDKNA gene deletion. In type A thymomas that lacked CDKN2A deletions and type B3 thymomas that rarely (7 %) exhibited CDKN2A deletion a different mechanism must predominate. CDKN2A promoter methylation is a known alternative mechanism of p16^I INK4A^ silencing and may dominate in type A and B3 thymomas.

PD-L1 is a molecule that binds to its receptor PD1, which is expressed on cytotoxic T-cells and exerts an inhibitory effect [59, 60]. PD-L1 is produced by a fraction of tumors of different entities and facilitates their immune escape [59, 60]. The blockade of the PD-L1/PD-1 interaction with anti-PD1 and anti-PD-L1 antibodies is a novel therapy with impressive results in various tumor entities [61]. In our study PD-L1 was expressed by only 13 % of type A thymomas, but 76 % of B3 thymomas and 53 % of thymic carcinomas. In contrast, a previous report by Padda et al. [62] that used a different anti-PD-L1 antibody, described PD-L1 expression in all cases of TETs. Furthermore, PD-L1 high TETs were associated with a more aggressive histology and worse prognosis. However, we did not observe a difference in survival between PD-L1 positive and negative thymic carcinomas. A lack of a difference in survival between PD-L1 positive and negative TETs has also been described by Katsuya et al. [63] Considering the correlation of PD-L1 expression by tumor cells with the likelihood of response to anti-PD-1/PD-L1 therapy [64], immune checkpoint inhibitors might be a novel treatment option for unresectable or relapsed thymomas and thymic carcinomas.

The expression of PTEN, a phosphatase that negatively regulates intracellular levels of phosphatidylinositol-3,4,5-trisphosphate and functions as a tumor suppressor by negatively regulating AKT/mTOR signaling pathways [65], was heterogenous, with no significant differences between type A and B3 thymomas and thymic carcinomas. A lack of PTEN expression was noted in only one B3 thymoma and one thymic carcinoma. However, both tumors retained two PTEN signals as assesed by FISH. Thus, the lack of PTEN expression may be caused by an alternative mechanism, like PTEN promoter methylation [66].

ROS1 is a tyrosine kinase that is aberrantly activated by translocation in a subset of lung carcinomas and cholangiocarcinomas. ROS1 translocated tumors frequently respond to therapy with crizotinib [67]. ROS1 positivity by immunohistochemistry is a surrogate marker for the presence of a ROS1 translocation. A weak ROS1 reactivity by immunohistochemistry was noted in three (20 %) type A thymomas, however, they did not harbor a ROS1 translocation as assessed by FISH. Type B3 thymomas and thymic carcinomas did not express ROS1. Therefore, ROS1 does not represent a therapeutic target in TETs.

In summary, our data show genetic differences between type A and B3 thymomas and thymic carcinomas with respect to cancer gene mutations and miRNA expression. The study furthermore demonstrates that next-generation gene panel sequencing of paraffin embedded tissue, which currently enters diagnostic pathology, combined with FISH and immunohistochemistry can identify potential novel therapeutic targets for TETs.

## Electronic supplementary material

Below is the link to the electronic supplementary material.

Supplementary Fig. S1 Overall survival of patients with thymic carcinomas with a normal or mutated (= mutated and/or deleted) CDKN2A gene (PDF 119 kb)

Supplementary Fig. S2 Overall survival of patients with thymic carcinomas with a normal or mutated (= mutated and/or deleted) TP53 gene (PDF 123 kb)

Supplementary Fig. S3 PDGFRA immunohistochemistry with low expression in a type A thymoma (a) and high expression in a thymic carcinoma (b). Original magnification x400(PDF 2457 kb)

Supplementary Fig. S4 PD-L1 immunohistochemistry with absent expression in a type A thymoma (a) and expression in a thymic carcinoma (b). Original magnification x400(PDF 1990 kb)

Supplementary Fig. S5 Overall survival of patients with thymic carcinomas negative or positive for PD-L1 protein expession (PDF 122 kb)

Supplementary Fig. S6 Overall survival of patients with thymic carcinomas negative or positive for p16^INK4A^ protein expession (PDF 88 kb)
