# Supplementary figures and images for: Molecular Profiling of Thymoma and Thymic Carcinoma: Genetic Differences and Potential Novel Therapeutic Targets
Source: Pathol Oncol Res. 2016 Nov 14;23(3):551–64. doi: 10.1007/s12253-016-0144-8 (PMC5487866; doi:10.1007/s12253-016-0144-8)

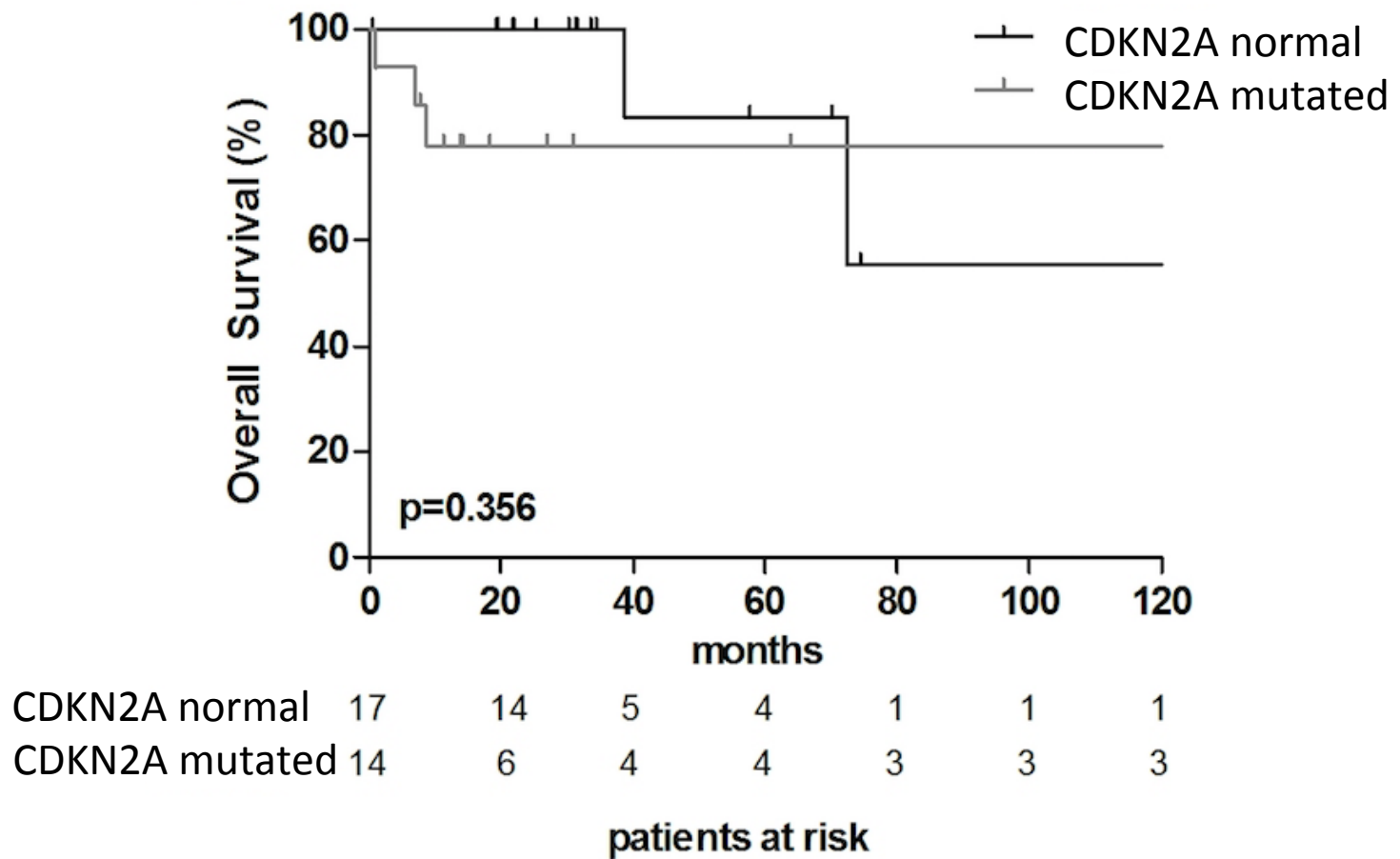

**Supplementary Fig. 1** Enkner F. *et. al.*

Supplement: Supplementary file 1 — Overall survival of patients with thymic carcinomas with a normal or mutated (= mutated and/or deleted) CDKN2A gene (PDF 119 kb) [file 12253_2016_144_MOESM1_ESM.pdf]

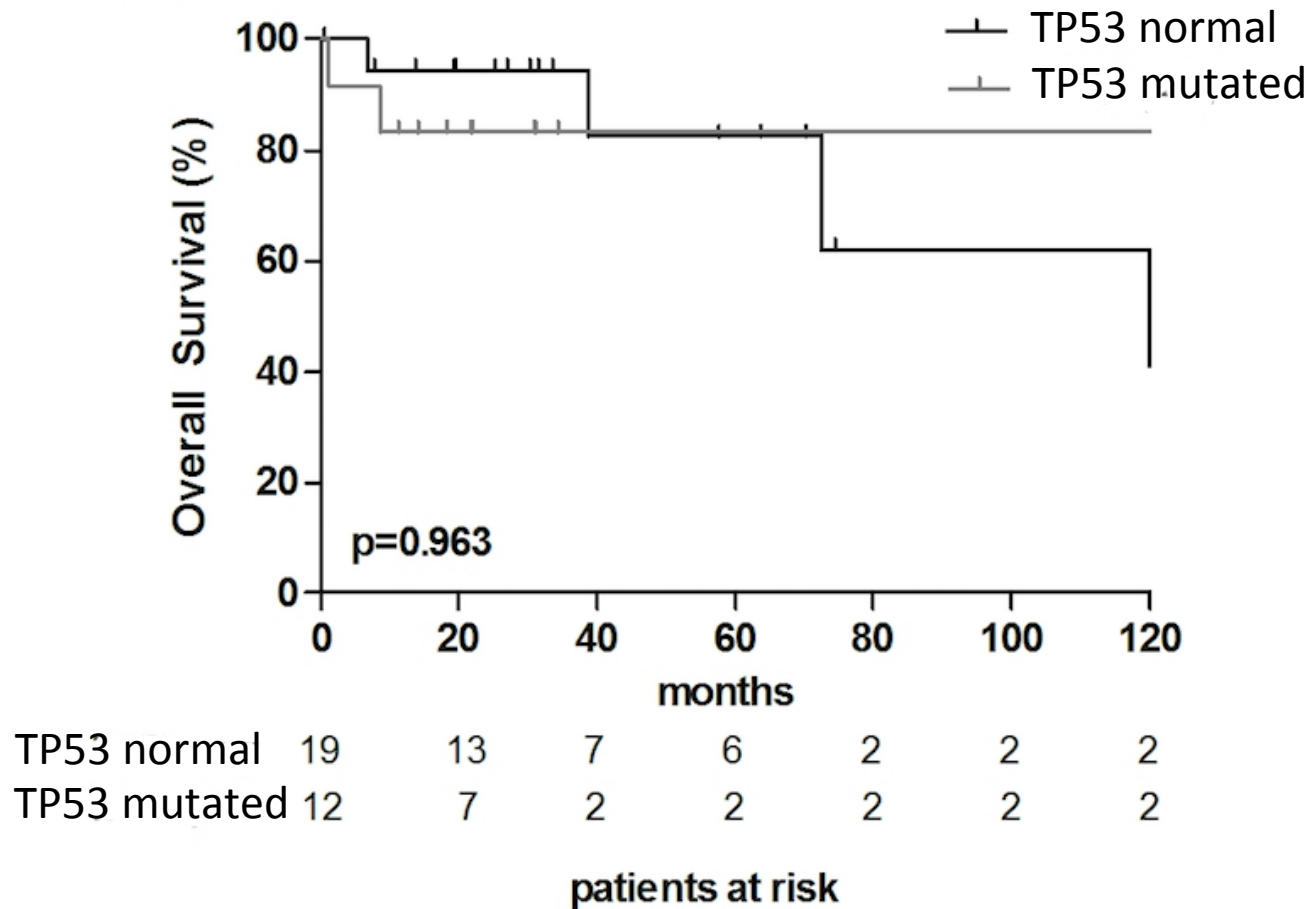

**Supplementary Fig. 2** Enkner F. *et. al.*

Supplement: Supplementary file 2 — Overall survival of patients with thymic carcinomas with a normal or mutated (= mutated and/or deleted) TP53 gene (PDF 123 kb) [file 12253_2016_144_MOESM2_ESM.pdf]

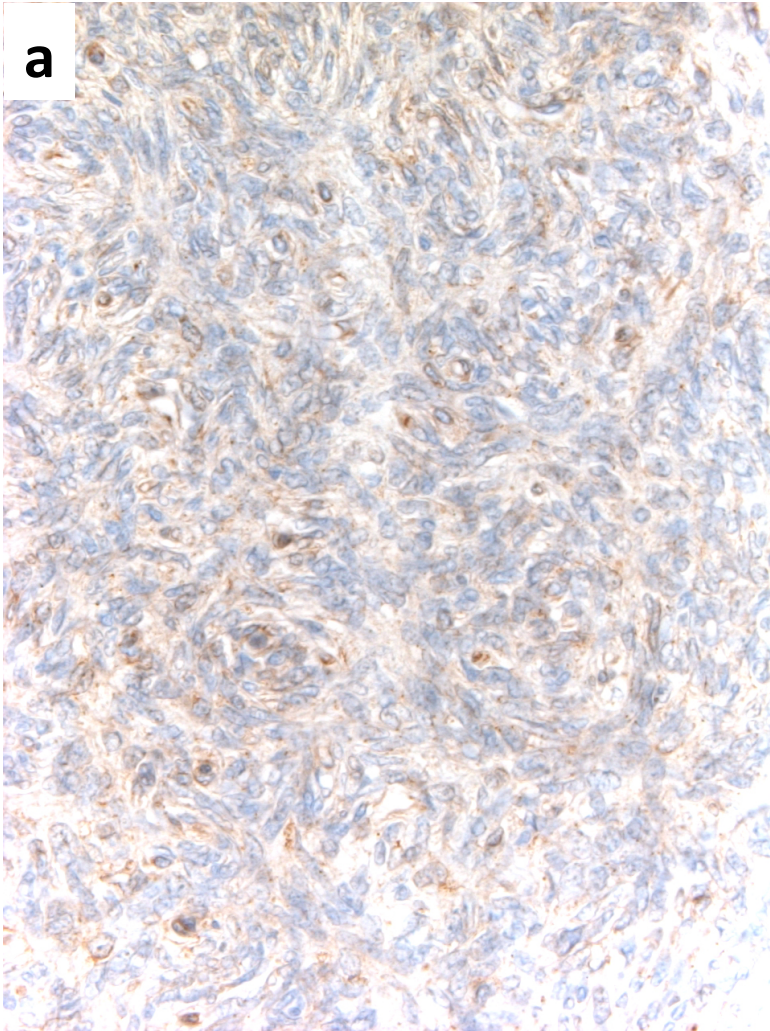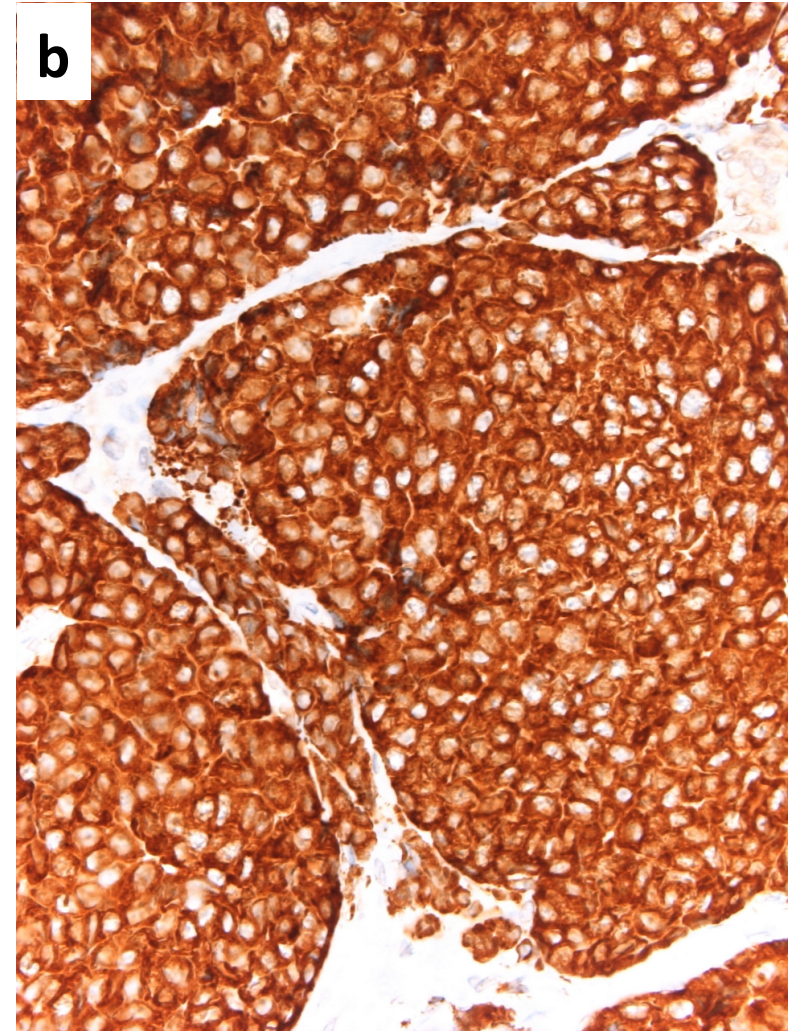

**Supplementary Fig. 3** Enkner F. *et al.*

Supplement: Supplementary file 3 — PDGFRA immunohistochemistry with low expression in a type A thymoma (a) and high expression in a thymic carcinoma (b). Original magnification x400(PDF 2457 kb) [file 12253_2016_144_MOESM3_ESM.pdf]

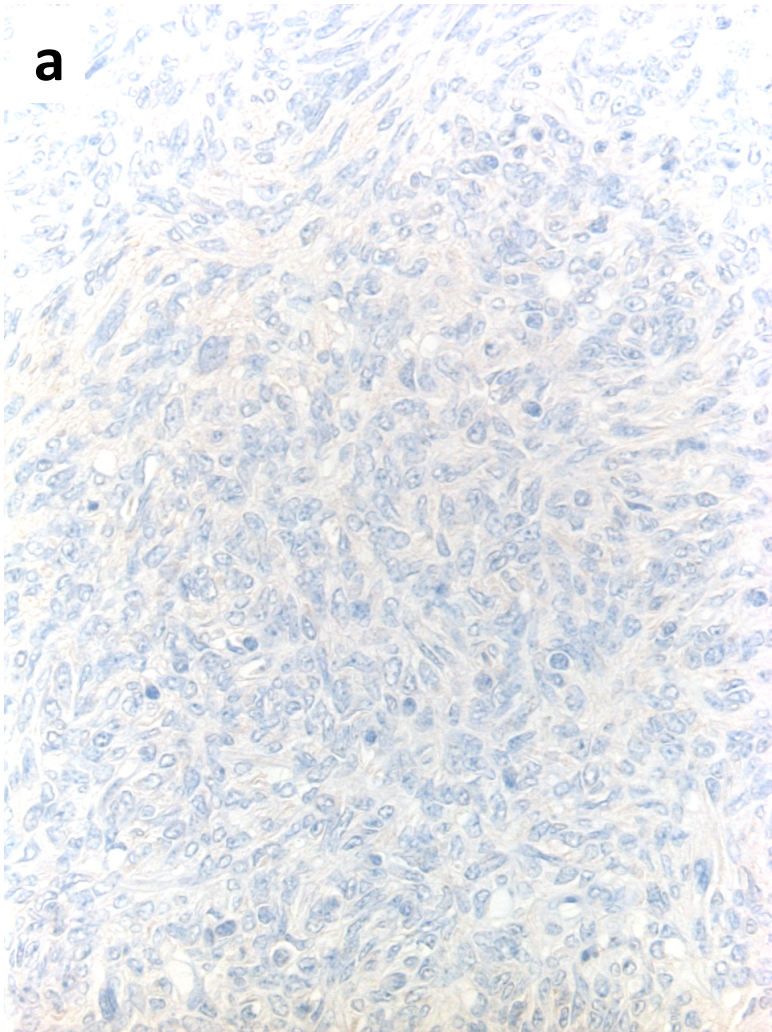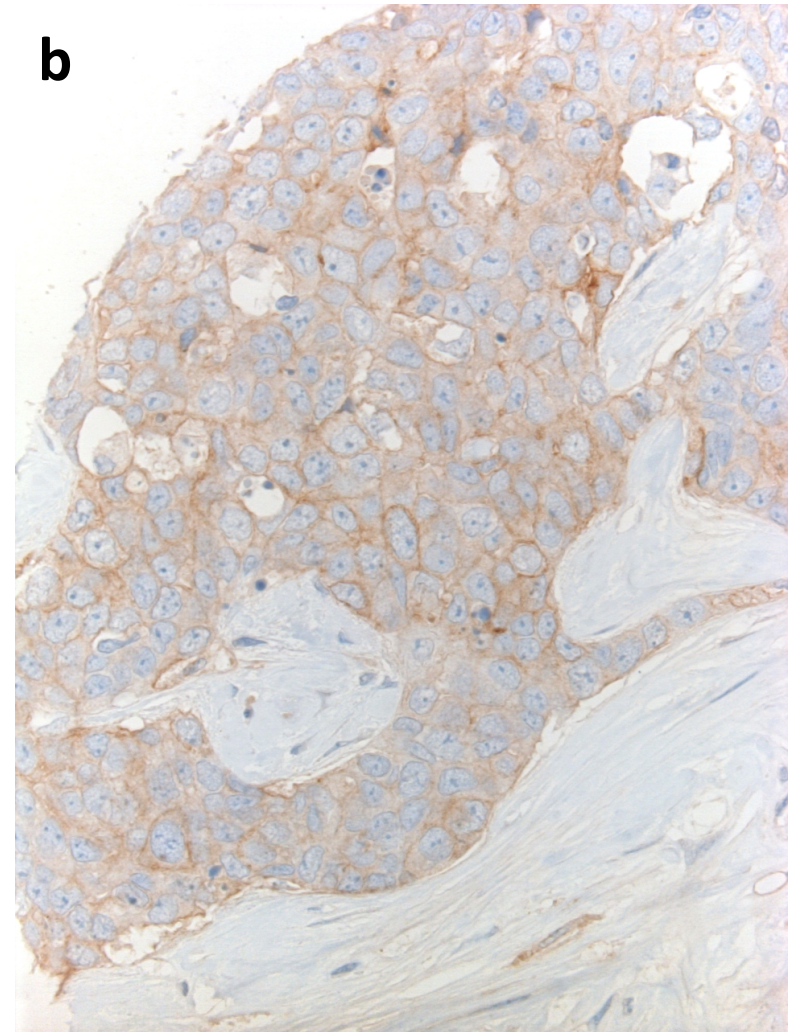

**Supplementary Fig. 4** Enkner F. *et al.*

Supplement: Supplementary file 4 — PD-L1 immunohistochemistry with absent expression in a type A thymoma (a) and expression in a thymic carcinoma (b). Original magnification x400(PDF 1990 kb) [file 12253_2016_144_MOESM4_ESM.pdf]

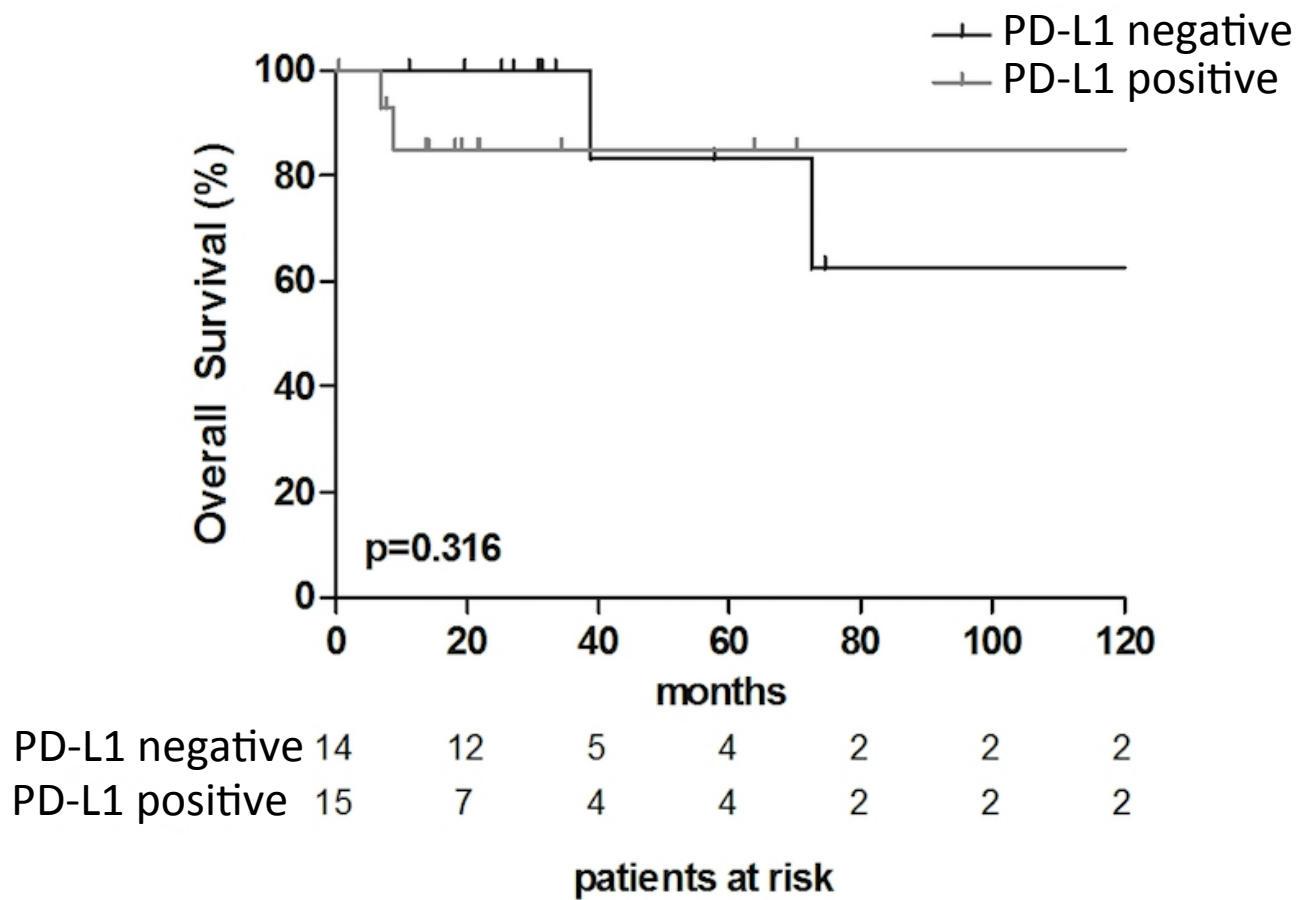

**Supplementary Fig. 5** Enkner F. *et. al.*

Supplement: Supplementary file 5 — Overall survival of patients with thymic carcinomas negative or positive for PD-L1 protein expession (PDF 122 kb) [file 12253_2016_144_MOESM5_ESM.pdf]

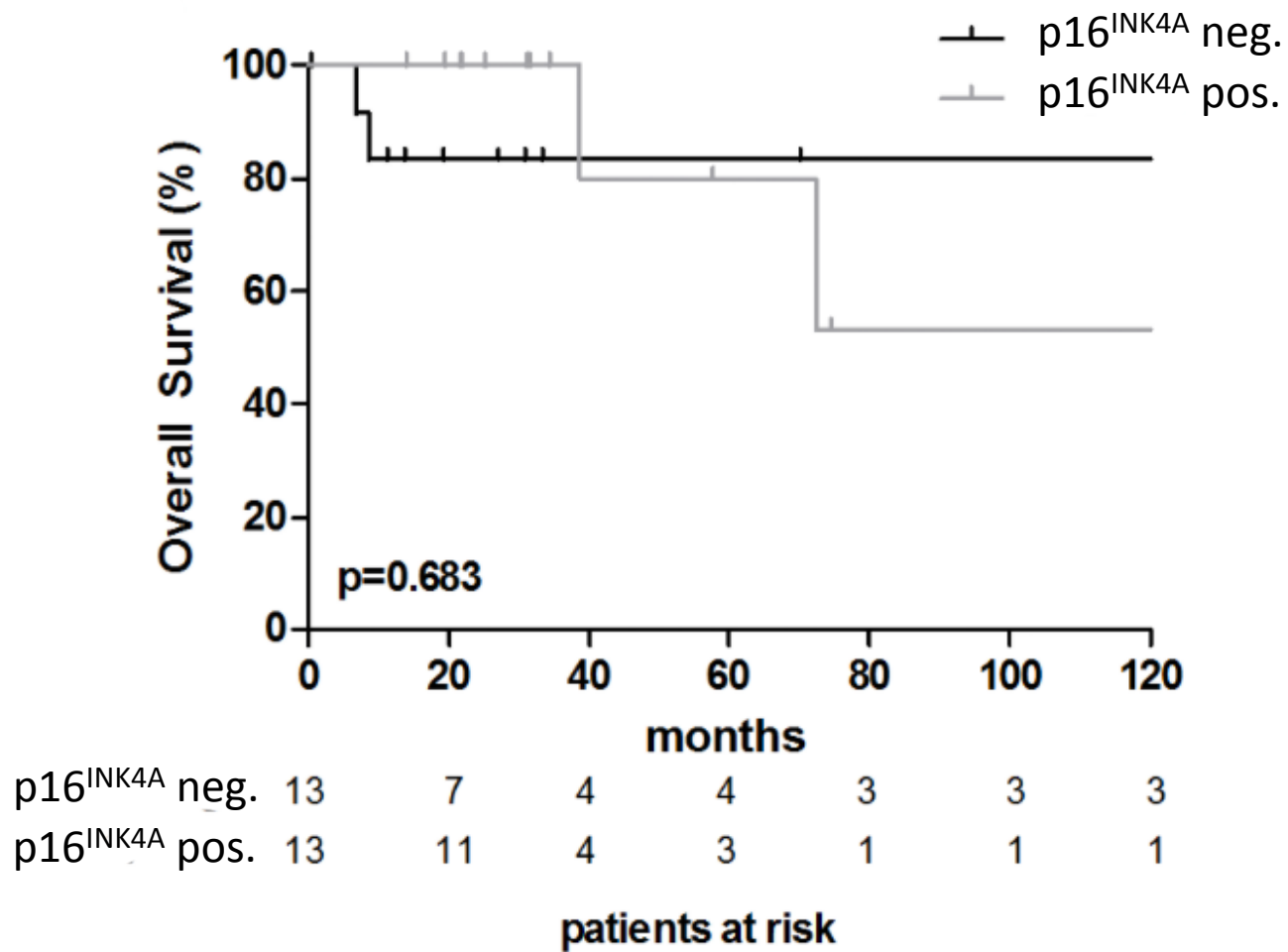

**Supplementary Fig. 6** Enkner F. *et al.*

Supplement: Supplementary file 6 — Overall survival of patients with thymic carcinomas negative or positive for p16INK4A protein expession (PDF 88 kb) [file 12253_2016_144_MOESM6_ESM.pdf]
